# Epicardial adipose tissue volume is independently associated with aortic root size as assessed by cardiac magnetic resonance imaging

**DOI:** 10.1186/1532-429X-17-S1-P372

**Published:** 2015-02-03

**Authors:** Bassel Sayegh, Rajeev R Fernando, Mushabbar A Syed, David J Wilber, Sanjay Singh, Tonye Teme, Mark G Rabbat

**Affiliations:** 1 Department of Cardiovascular Medicine, Loyola University Medical Center, Maywood, IL, USA; 2 Department of Biological Sciences, East-West University, Chicago, IL, USA; 3 Department of Internal Medicine, Loyola University Medical Center, Maywood, IL, USA

## Background

Epicardial adipose tissue (EAT), located between the myo-epicardium and the visceral pericardium, is a metabolically active organ and secretes pro-inflammatory, fibrotic and atherogenic adipokines. The absence of a fascial layer between EAT and aortic adventitia may allow the EAT secretome to alter aortic extracellular matrix promoting aortic root dilatation. Aortic root size has been shown to be an independent predictor of incident heart failure, stroke, and cardiovascular and all-cause mortality. The aim of this study was to assess the association of EAT volume with aortic measurements both within and outside the pericardium using cardiac magnetic resonance imaging (CMR).

## Methods

We retrospectively studied 49 patients with atrial fibrillation who underwent CMR prior to ablation (Table 1). CMR Images were acquired on a 3T scanner (Siemens Trio) by steady state free precession (SSFP) using a standard short axis stack through the atria and ventricles. EAT was assessed volumetrically in consecutive short-axis views through the atria and ventricles using the multi-slice method during end-diastole. Descending thoracic aorta measurements were obtained in the static axial SSFP images at the level of the pulmonary artery bifurcation. Maximum linear dimensions were used for analysis. Aortic root size was measured at end-diastole from the 3 chamber view at the level of the sinus of Valsalva.

## Results

Patients with an aortic root ≥ 3.7 cm had significantly higher EAT compared to those with an aortic root < 3.7 cm (152 ± 71 ml vs 113 ± 57 ml, p<0.001). Patients with EAT ≥ 110 ml had significantly larger aortic root not descending thoracic aorta size compared to EAT < 110ml (3.4 ± 0.3 cm vs 3.2 ± 0.4 cm, p<0.03 and 2.5 ± 0.3 cm vs 2.4 ± 0.3 cm, p<0.08 respectively). By univariate analysis, EAT volume was significantly correlated with aortic root size (r = 0.48, p=0.02), descending thoracic aorta size (r = 0.29, p=0.04) and BMI (r = 0.44, p=0.002) but not hypertension (r = 0.25, p=0.09). EAT volume remained the only significant independent predictor of aortic root size but not descending thoracic aorta size in a multivariate linear regression model incorporating age, BMI, and hypertension (p=0.007).

## Conclusions

EAT volume is predictive of aortic root not descending thoracic aorta size independent of traditional risk factors. These findings suggest that the secretome of EAT may play a local causative role in the pathogenesis of aortic root dilatation.

## Funding

None.

**Table 1:** Baseline patient characteristics

Parameters	Patients (n=49)
Age (years)	59.52 ± 9.74

Male	31 (63)

Diabetes mellitus	4 (8)

Hypertension	23 (47)

Obstructive sleep apnea	3 (6)

Paroxysmal atrial fibrillation	40 (81)

Body mass index (kg/m2)	28.34 ± 5.96

Epicardial adipose tissue volume (mL)	117.39 ± 59.58

Aortic root diameter (cm)	3.3 ± 0.35

Descending aorta diameter (cm)	2.5 ± 0.27

Data are expressed as means ± standard deviation or number (%) of patients

